# In this issue: Volume 113, Issue 7, July 2022

**DOI:** 10.1111/cas.14956

**Published:** 2022-07-13

## IL‐27 improves adoptive CD8^+^ T cells’ antitumor activity via enhancing cell survival and memory T cell differentiation


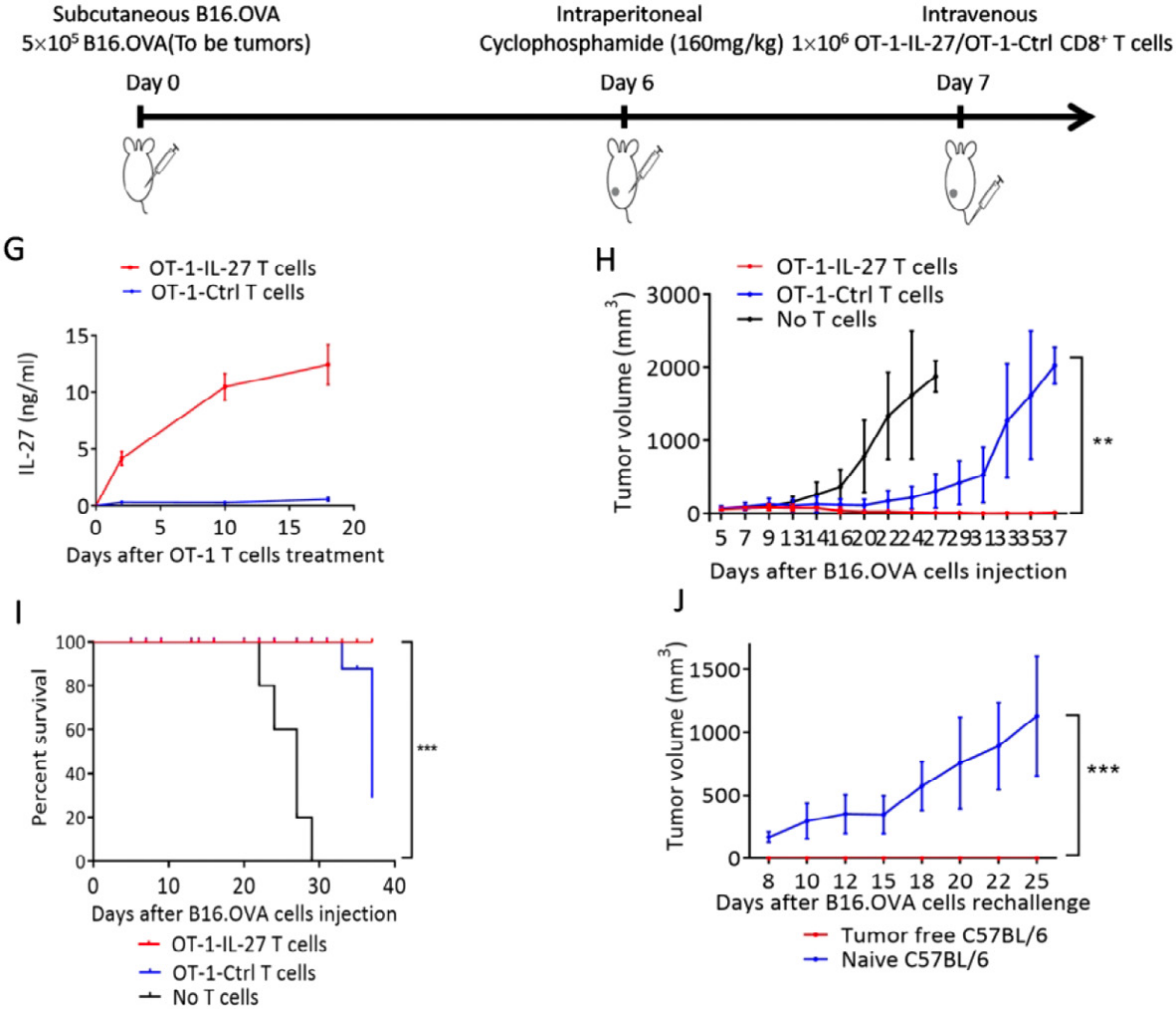


Adoptive T‐cell therapy is a type of cancer treatment in which T cells, which are a type of immune cells, are used to treat patients with cancer. Often, these T cells are genetically modified to recognize and kill tumor cells and have shown promising treatment outcomes against cancer. However, maintaining this anti‐tumor response of the adoptive T cells continuously over long periods of time has proven to be difficult.

Recent observations show that immune molecules known as cytokines display strong antitumor activity. Interleukin‐27 (IL‐27) is one such cytokine, which has shown high efficacy against multiple types of tumors.

In this issue, Ding et al. tried to understand if IL‐27 can act as an ‘adjutant’, or a supporter, to CD8^+^ T cells—a subtype of T cells with cytotoxic or cell‐killing properties—as part of adoptive therapy against cancer. To this end, they genetically modified CD8^+^ T cells and tested their anti‐tumor efficacy with and without IL‐27, on lab‐cultured tumor cells, and in tumor bearing mice.

They noted that IL‐27 increased the activation of CD8^+^ T cells and enhanced their tumor cell‐killing activity. Importantly, it increased the survival time of CD8^+^ T cells in cell cultures and in tumor‐bearing mice, a property which is crucial for an improved anti‐tumor response. In fact, IL‐27 also increased the expression of genes that program CD8^+^ T cells to act like memory T cells, i.e., cells that remember an infection and how to respond against it for a long time. The resultant memory T cells in this case had the added ability to respond rapidly and effectively upon encountering tumor cells again. Additionally, the authors modified CD8^+^ T cells to deliver IL‐27 in tumor bearing mice and observed a complete regression of these tumors. This regression indicates that IL‐27 delivered through CD8^+^ T cells improves their immunity against tumor cells by a large extent.

These findings suggest that IL‐27 can be used as a suitable adjutant for T cells and can improve the treatment outcomes for patients undergoing adoptive T cell therapy for cancer.

https://onlinelibrary.wiley.com/doi/10.1111/cas.15374

## Extracellular ATP promotes angiogenesis and adhesion of TNBC cells to endothelial cells via upregulation of CTGF


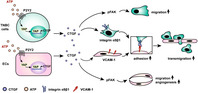
Breast cancer is one of the most common types of cancer affecting women, and a leading cause of death worldwide. Several of these deaths are caused by the metastasis of the breast cancer cells, i.e., their migration from the breast tissue to other parts of the body. Triple‐negative breast cancer (TNBC) is a severe form of breast cancer that is particularly prone to metastasis and results in poor patient outcomes.

It has been observed that ATP, a molecule that provides energy to cells, is found in increased quantities in the microenvironment where tumors proliferate. It enables the migration and invasion of these tumors into different tissues and organs. Targeting the molecular factors that promote TNBC metastasis could help in treating this cancer, but these factors are yet to be identified.

In this issue, Zhou et al. examined how extracellular stores of ATP affected the molecular mechanisms responsible for TNBC metastasis. They focused on two types of cells: TNBC cells, which are cancerous and can metastasize, and endothelial cells (ECs), which support metastasis by forming blood vessels and supplying nutrients to metastatic tumors.

Zhou et al. observed that in both TNBC cells and ECs, extracellular ATP increased the expression (or production) of a molecule known as connective tissue growth factor (CTGF), which in turn increased the formation of blood vessels required for tumor growth. ATP also increased the interaction between TNBC cells and ECs, which enabled the migration of TNBC cells through tissues formed of ECs.

The authors tested the validity of these findings in mice containing TNBC cells. On treating these mice with inhibitors of ATP and CTGF, they observed a reduction in the metastasis of tumor cells. Notably, tumors treated with these inhibitors also displayed reduced blood vessel formation.

Together, these findings show that extracellular ATP increases formation of tumor blood vessels, and the interaction between TNBC cells and ECs, by increasing the expression of CTGF. Therefore, the development of therapies that target extracellular ATP and CTGF may prevent TNBC metastasis and improve patient outcomes worldwide.

https://onlinelibrary.wiley.com/doi/10.1111/cas.15375

## Opposing roles of HDAC6 in liver regeneration and hepatocarcinogenesis


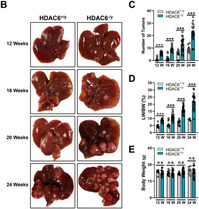


Hepatocellular carcinoma (HCC) or liver cancer is one of the most common causes of cancer‐related deaths worldwide. It typically develops in patients with persistent liver injury, i.e., permanent damage to the liver cells, which usually occurs due to chronic liver diseases, suggesting the complex interaction between cancer and immune system.

Recently, histone deacetylase 6 (HDAC6) has emerged as a privileged inhibitory target for cancer therapy because of its deacetylating activity for p53 at K120. However, the intricate roles of HDAC6 in hepatocellular carcinogenesis have been suggested by recent evidence that HDAC ablation suppresses innate immunity, which plays critical roles in tumor immunosurveillance and antitumor immune responses. Therefore, it is important to determine whether HDAC6 ablation inhibits liver regeneration and hepatocellular carcinogenesis using *in vivo* animal models.

In this report, Sophors et al. showed that HDAC6 ablation increased K320 acetylation of p53, also known as pro‐survival acetylation, in all tested cellular and animal models, but did not always increase K120 and K373/382 acetylation of p53, also known as pro‐apoptotic acetylation. Despite HCC was promoted by the genetic ablation of HDAC6, the proliferation of cancerous hepatocytes was significantly reduced in HCC mice model. In other word, liver cancer cells itself slowly grow, but liver cancer grow fast in HDAC6 knockout mice. This paradoxical observation leads to astonishing finding that antitumoral innate immune activity was compromised in HDAC6 knockout mice, which is sufficient to get tumoral outgrowth despite of reduce cancer cell growth itself.

In summary, Sophors et al. provides the first evidence that HDAC6 is a p53 deacetylase at K320, which is especially important for cancer cell survival in chronic DNA damage conditions. Contrary to the general assumption that HDAC6 inhibition leads to hyperacetylation of p53 at K120, resulting in tumor suppression, their findings from in vivo animal HCC models emphasize the importance of the opposing roles of HDAC6 ablation in hepatocellular carcinogenesis by highlighting the K320 acetylation of p53 and immunosuppressive effects.

https://onlinelibrary.wiley.com/doi/10.1111/cas.15391

